# Liver Transplantation for Acute Liver Failure Attributed to Leptospirosis: A Report of Two Cases

**DOI:** 10.1155/2019/5189542

**Published:** 2019-12-17

**Authors:** Thibault Lebreton, Fréderic Aubrun, Jean-Yves Mabrut, Laurent Heyer, Camille Perrin

**Affiliations:** ^1^Hospices Civils de Lyon, Hôpital de la Croix-Rousse, Service d'Anesthésie Réanimation, 103 Grande Rue de la Croix-Rousse, 69004 Lyon Cedex, France; ^2^Hospices Civils de Lyon, Hôpital de la Croix-Rousse, Service de Chirurgie Générale, Digestive et Transplantation Hépatique et Intestinale, 103 Grande Rue de la Croix-Rousse, 69004 Lyon Cedex, France

## Abstract

**Background:**

Leptospirosis is a zoonosis caused by pathogenic spirochetes of the genus Leptospira. Although it may be limited to nonspecific fever, leptospirosis may also be responsible for neurological symptoms or fulminant diseases such as Weil's disease. Diagnosis is challenging due to the difficulty in isolating the organism and the delays required for performing the serological test.

**Case Presentation:**

Two cases of leptospirosis are presented here. The clinical picture differed from a real Weil's disease in the first case and from a neuro-leptospirosis in the second. However, both patients underwent liver transplantation because of the severity of the associated acute liver failure. Unfortunately, one of the cases had a fatal outcome.

**Conclusion:**

Antibiotic treatment for leptospirosis should not be delayed by the lack of a positive serology test for this potentially lethal disease. In the context of a history of exposure to risk factors for leptospirosis, a negative serology must be repeated 7 days to 2 weeks following the first test. Although not always present, acute liver injury may, in rare cases, require liver transplantation.

## 1. Introduction

It is estimated that more than 1 million severe cases of leptospirosis occur each year worldwide, with a mortality rate of 5–20% [1]. Leptospires are bacteria that colonise renal tubules of a large number of wild animals (rodents), and domestic animals (cattle, pigs, dogs) before being excreted in urine. The mode of transmission to humans can be direct (contact with infected animals), or indirect (through water and soils contaminated by infected urine) [2].

Leptospirosis demonstrates diverse clinical presentations, ranging from the anicteric febrile form, observed in the vast majority of cases, to extensive tissue damage, vasculitis that may be responsible for neurological manifestations and multiorgan failure. We report two cases of severe forms of leptospirosis with different clinical presentations that both required liver transplantation.

## 2. Case 1

A 23-year-old man was admitted to the Emergency Department following a 5-day history of fever with myalgia in calf muscles, jaundice, a purpuric rash on his feet, haematemesis, and anuria for 2 days.

There was no medical, surgical, or contributory medical history. The patient did not drink alcohol, smoke, or use illicit drugs. There was no travel history, but the patient reported fishing in rivers regularly and had been bitten by a ferret in the 15 days preceding presentation. Laboratory findings in the emergency ward were: haemoglobin 80 g/L, white blood cell count (WBC) 22 × 109 cells/L (neutrophils 90%), platelet count 13 × 10^9^ cells/L, aspartate aminotransferase (AST) 174 IU/L, alanine aminotransferase (ALT) 110 IU/L, total bilirubin 28 mg/dL, international normalised ratio (INR) 2.7, serum creatinine 6 mg/dL, serum urea 106 mg/dL.

Antibiotic therapy with ceftriaxone and spiramycin was initially started after computed tomography (CT) scan of the thorax and abdomen showed diffuse micronodular interstitial syndrome. The patient was transferred to the intensive care unit due to acute liver failure and anuria that required continuous venous haemafiltration, (CVVH) therapy.

Hepatitis A, hepatitis B, and hepatitis C viruses were excluded by hepatitis-virus-panel investigation. There was no laboratory evidence of infection with Epstein-Barr virus, or Cytomegalovirus. Serum paracetamol concentration was less than 1 mg/L. Ceruloplasmin, *α*-1-antitrypsin, ferritin and an autoimmune screen were all unremarkable.

Based on the epidemiological context, compatible symptoms and biological results, an icterohaemorrhagic leptospirosis (Weil's disease) was suspected. This diagnosis was rapidly confirmed by positive leptospira serology combining IgM enzyme-linked immunosorbent assay method (ELISA), and microscopic agglutination test (MAT), as well as quantitative polymerase chain reaction (PCR) of blood and urine performed 7 days after the onset of symptoms. The patient was then treated with ceftriaxone alone for 2 weeks.

In the following days, the patient continued to have haematemesis and epistaxis. Liver involvement was mainly marked by cholestasis, with a maximum of total bilirubin at 65.9 mg/dL, AST at 524 IU/L, and ALT at 432 IU/L.

On the 10th day, the patient experienced haemorrhagic shock caused by bleeding from the pancreaticoduodenal artery. The patient had an asymptomatic median arcuate ligament syndrome, responsible for the development of numerous collateral fragile arteries. The haemorrhagic shock was controlled by embolisation of the pancreaticoduodenal artery.

Whilst the situation was stable, despite the persistence of liver and kidney failure, a gastroscopy was performed on the 30th day due to the appearance of melena. It found a complete ischemic necrosis of the duodenum with perforation, which was treated with a cephalic duodenopancreatectomy.

A deterioration was then noted, with major cytolysis and rapid development of acute liver failure (AST 16650 IU/L, ALT 7900 IU/L, total bilirubin 17 mg/dL, INR 3.12), requiring urgent orthotopic liver transplantation only 2 days later. Despite liver transplantation, the patient died 3 days after transplantation (36th day of hospitalisation) due to multiorgan failure and primary liver graft dysfunction.

## 3. Case 2

A previously healthy 60-year-old man, presented with fever, chills, and sweats, followed by an altered level of consciousness with a Glasgow coma scale score at 3. He had returned 18 days earlier from a trip to India and underwent a transrectal ultrasound guided prostate biopsy with ciprofloxacin antibiotic prophylaxis the day before. The patient was then sedated, intubated, and admitted to intensive care. The pupils were equal and he had no nuchal rigidity. Initial laboratory examination of peripheral blood showed: haemoglobin 158 g/L, white blood cell count (WBC) 13 × 109 cells/L (neutrophils 90%), platelet count 80 × 10^9^ cells/L, AST 605 IU/L, ALT 355 IU/L, total bilirubin 3.4 mg/dL, INR 3.12, serum creatinine 2.2 mg/dL, serum urea 28 mg/dL.

Management consisted in invasive mechanical ventilation, norepinephrine infusion, and anti-infective treatment with cefotaxime, amoxicillin, and aciclovir, quickly replaced by meropenem, fosfomycin, and amikacin following the discovery of a multidrug-resistant Escherichia Coli on blood cultures.

Despite stopping the sedative infusions and a normal brain CT scan, there was no change in the level of consciousness. Furthermore, peripheral blood analysis demonstrated rapidly deteriorating acute kidney and liver failure:haemoglobin 110 g/L, white blood cell count (WBC) 15 × 109 cells/L (neutrophils 90%), platelet count 17 × 10^9^ cells/L, AST 17000 IU/L, ALT 9500 IU/L, total bilirubin 4.7 mg/dL, INR 6.76, serum creatinine 4.5 mg/dL, serum urea 36 mg/dL.

A liver screen including serological testing for hepatitis A, B, C, HIV, herpes simplex virus, cytomegalovirus, and Epstein-Barr virus (EBV) was negative. Serum paracetamol concentration was less than 1 mg/L. Ceruloplasmin, *α*-1-antitrypsin, ferritin and an autoimmune screen were all negative. Dengue and other arbovirus infections were ruled out, and thick blood films were negative for malaria parasites. The initial leptospirosis serology (ELISA and MAT) was negative. Abdominal ultrasound did not demonstrate any vascular cause.

Renal replacement therapy with CVVH and hepatoprotection (*N*-acetylcysteine) were initiated. Finally, a highly urgent orthotopic liver transplantation was performed for fulminant hepatic failure, 6 days after the initial appearance of symptoms.

Following liver transplantation and cessation of sedation, whilst liver function tests and serum ammonia level returned to normal, an altered level of consciousness persisted which was associated with cerebellar ataxia, facial dyskinesia, swallowing disorders, and tremulousness of hands. Electroencephalography, cranial magnetic resonance imaging, and cerebrospinal fluid (CSF) analysis (no cells; normal glucose and proteins) were unremarkable. The diagnosis of leptospirosis with fulminant hepatitis failure and neuro-leptospirosis was finally made in view of the positivity of leptospira serology (ELISA and MAT) 2 weeks after the onset of symptoms. An Indian strain of Leptospira Hebdomadis was identified. The interrogation taught us later that the patient was led into a river whilst on an elephant ride, 3 days before his return to France. PCR performed 25 days after admission did not detect Leptospira DNA in the blood, CSF, or urine.

The clinical evolution was finally favourable, with progressive disappearance of cerebellar ataxia and improved renal function after several weeks. The patient was discharged to return home 74 days after the onset of symptoms.

## 4. Discussion

France, with an annual incidence between 0.5 and 1 case/100.000 inhabitants, is one of the industrialised countries with the highest incidence [3]. In nonendemic areas, travel is the main cause of leptospirosis. Other described risk factors are aquatic activities in potentially contaminated fresh water and some exposed professions: farmers, slaughterhouse employees, sewer workers, and gardeners. After an incubation period of 3–30 days, human leptospirosis may have diverse clinical manifestations making its diagnosis difficult. Icterohaemorrhagic leptospirosis or Weil's disease (including the major triad of acute renal failure, jaundice, and haemorrhagic diathesis) is the most severe form of the disease which carries a significant risk of mortality. Despite presenting with different symptoms, patients in the presented cases had acute liver failure requiring urgent orthotopic liver transplantation.

The first patient developed a fulminant febrile illness with jaundice, myalgia in calf muscles, acute kidney injury, and significant gastrointestinal bleeding, probably due, in part, by a median arcuate ligament syndrome. Serum bilirubin in Weil's disease is often markedly elevated with moderate elevation of transaminases and alkaline phosphatase [4]. Such findings were evident in this case, whereby serum transaminases were initially slightly raised, with a marked increase in total bilirubin which was greater than 60 mg/dL. Serum transaminase levels began to increase following arterial embolisation and cephalic duodenopancreatectomy. It is likely that the median arcuate ligament syndrome played a major role in the gastrointestinal bleeding and subsequent complications which led to the patient's death. Many collateral arteries developed to compensate for the reduced celiac artery blood flow due to extraluminal compression by the medial arcuate ligament. The vasculitis, due to leptospirosis infection, likely caused the bleeding on these already fragile collateral arteries.

For the second case, the initial symptomatology was less typical as the patient presented sudden neurological manifestations with an altered level of consciousness followed by cerebellar ataxia. The severity of the associated acute liver failure required liver transplantation. Neuro-leptospirosis occurs in around 10–15% of patients [5]. Aseptic meningoencephalitis is the most common neurological manifestation [6], but cases of myeloradiculopathy, myelopathy, and Guillain Barre syndrome have also been reported. Cerebellar involvement has also been described and is thought to affect less than 5% of cases [7]. Neurologic involvement in leptospirosis is due to capillary endothelial damage and vasculitis, and its prognosis is generally good. It is difficult to determine whether the prostate biopsy, performed the day before onset of symptoms, played a role in the clinical severity of this case. It is even more intriguing since the patient was administered ciprofloxacinas antibiotic prophylaxis, which is usually effective in leptospirosis infections.

Liver damage is common in leptospirosis infection and can present as slight disturbances in serum liver function tests or fulminant hepatic failure. Previous reports concerning pathological findings of liver disease in leptospirosis include hyperplasia of Kupffer cells, cholestasis, cell infiltration in portal areas, and sometimes extensive necrosis of hepatocytes leading to hepatic failure [8]. This has been demonstrated in these two cases, where extensive hepatocyte necrosis was identified on histopathological investigation of explanted liver, after transplantation. As demonstrated through the cases presented herein, liver injury can be rapid, important, and irreversible, sometimes leading to the death of the patient.For this reason, even in the absence of encephalopathy, every patient with a severe acute hepatitis associated with leptospirosis infection should be referred to a hospital with a transplant centre for optimal clinical management, which may include transplantation. If liver transplantation is indicated, timing will be important.

**Figure 1:**
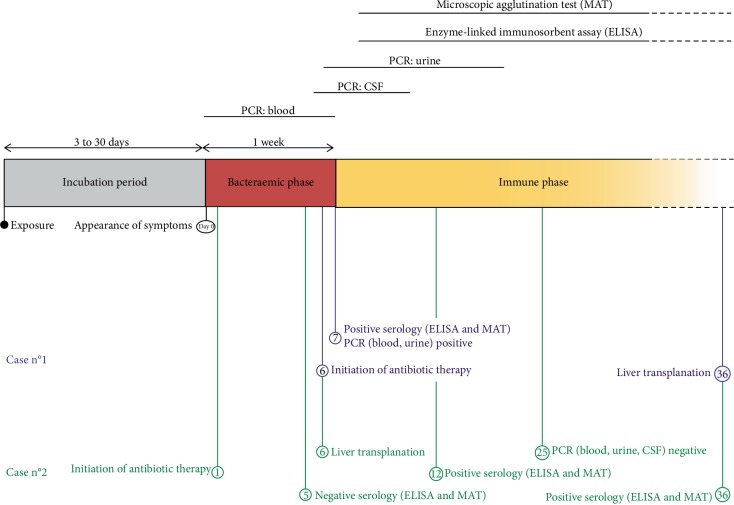
Timeline of leptospirosis management: diagnostic tests, clinical data, and test results for two cases.

Serology is the most common test used to diagnose leptospirosis. MAT is the gold standard to detect both immunoglobulin M (IgM) and immunoglobulin G class agglutinating antibodies. However, it requires significant expertise [9]. IgM ELISA are more commonly performed as an initial screening test as they are easier to carry out, although less specific. Interpretation of serology can be complicated as the first sample is negative in half of the cases, and the test can remain negative when early antibiotic treatment is administered [10]. Therefore, negative serology does not exclude the diagnosis and must be repeated 7 days to 2 weeks later. Molecular methods detect leptospira nucleic acids in the blood in the days following the onset of clinical signs (bacteraemic phase). Leptospires then disappear from the bloodstream (immune phase) (Figure 1). PCR can sometimes detect leptospira in the CSF, and transiently in the urine, from the second week of the pathological course. In the first case, leptospirosis diagnosis was made 7 days after the appearance of the first symptoms, by positive MAT and ELISA, confirmed with PCR detection of leptospira DNA in blood and urine.In the second case, reaching a diagnosis took longer. Indeed, a first leptospira serology test, performed during the first days of hospitalisation, was negative. The diagnosis was made 12 days later by a second positive serology (ELISA and MAT). Furthermore, PCR performed on blood, CSF, and urine samples more than 3 weeks after the first symptoms did not detect leptospira DNA. The value of the various diagnostic tests therefore depends on the stage of the disease and whether the patient has received antibiotics.

Early antibiotic treatment of leptospirosis has been associated with a better prognosis [11]. Intravenous penicillin is the drug of choice, although ceftriaxone, cefotaxime, and carbapenems are also effective. Treatment duration is 5–7 days. In case of allergy to penicillin, the treatment of choice is orally administered doxycycline. Importantly, the choice of antibiotic administered does not depend on the leptospira serotype.

In conclusion, Leptospirosis infection is characterised by a generalised vasculitis responsible for a broad spectrum of clinical manifestations, including acute organ failure that can lead to death. The two reported cases presented differing clinical pictures; however, both patients experienced significant liver injury that required orthotopic liver transplantation.
